# Age negatively impacts reproduction in high-ranking male rhesus macaques on Cayo Santiago, Puerto Rico

**DOI:** 10.1038/s41598-020-69922-y

**Published:** 2020-08-03

**Authors:** Krista M. Milich, Angelina Ruiz-Lambides, Elizabeth Maldonado, Dario Maestripieri

**Affiliations:** 1 Department of Anthropology, Washington University in St. Louis, St. Louis, MO USA; 2 Institute for Mind and Biology, University of Chicago, Chicago, IL USA; 3 Caribbean Primate Research Center, University of Puerto Rico, San Juan, Puerto Rico

**Keywords:** Animal behaviour, Ageing, Sexual selection

## Abstract

Based on sexual selection theory, the reproductive potential of male primates is expected to be limited by access to fertile females. Alpha males, the highest ranking males in a social group, are predicted to have better access to mates and produce more offspring until they are no longer dominant, which usually corresponds with age. Little is known about male reproductive senescence independent of rank changes in nonhuman primates. Here, we examine variation in the reproductive success of high-ranking male rhesus macaques on Cayo Santiago. We recorded behavioral data for 21 adult males across 9 social groups during the 2013 mating season. Additionally, we used paternity data from the long-term database to determine the number of offspring each subject sired over his lifetime and during the study period. Older high-ranking males in stable groups had fewer offspring than younger high-ranking males in stable groups in 2013. The low reproductive output for the older males was not a result of lower mating effort, and reproductive output in 2013 was not predicted by total prior reproductive success. Our results provide novel evidence of post-copulatory reproductive senescence in high-ranking male nonhuman primates.

## Introduction

Sexual selection theory predicts that male primates are mainly limited in their reproductive potential by access to fertile females^1^. Within nonhuman primate groups, alpha males (the highest-ranking male in each group) have better access to mates^2^ and are therefore expected to produce more offspring until they are no longer dominant (e.g. baboons^3^; mandrills^4^), which is usually associated with older age due to the energetic and physical demands of maintaining dominance status^5,6^. This pattern varies greatly between primate species depending on their social structure and grouping pattern. Rhesus macaques live in large multi-male, multi-female groups where both males and females form strong linear dominance hierarchies in which high-ranking individuals have better access to resources, including mating opportunities.

Relatively little is known about male reproductive senescence, or age-related declines in reproduction, independent of rank changes in primates, although a large body of research literature on this topic exists for human males^7–13^. In men, age has been negatively associated with sperm quality and positively associated with a risk of genetic disorders in offspring^9,12,14^ (but see^10^). Importantly, studies on men do not show consistent changes in hormone concentrations with age, suggesting that hormone concentrations are not a good predictor of reproductive function and should not be used as a proxy measure in primates^15,16^.

In nonhuman primates, the focus has primarily been on female reproductive senescence (e.g. rhesus macaques^17–19^), and many studies have simply assumed that alpha males who are seen mating frequently are producing offspring, regardless of their age^20^. As studies have started incorporating paternity data, it has become clear that mating success does not always equal reproductive success^21^. A few studies have found evidence of changes in sexual behaviors with age in male nonhuman primates, but there is limited evidence of a decrease in fertility (reviewed in^22^). The ability to study reproductive senescence in free-ranging primates is limited by the confounding factor that many older males lose their high-ranking status prior to a decrease in their reproductive success^5,23,24^. For example, male baboons experience a decline in reproductive output as they age, but this decline coincides with a loss in dominance rank^5^. In these cases, a decrease in reproductive success appears to be associated with reproductive senescence in pre-copulatory mechanisms; however, it does not address potential senescence in post-copulatory mechanisms, such as ejaculate quality. Males that are able to maintain mating opportunities with females throughout their life may still experience senescence in the ability to fertilize an egg or produce a viable offspring^25^.

The population of rhesus macaques (*Macaca mulatta*) on Cayo Santiago, Puerto Rico^26^, provides a unique opportunity to examine the impact of age on male reproductive output for several reasons. First, the long-term database for this population includes the age of each individual and detailed records of how many offspring each male has sired over his lifetime. Second, the influence of socio-demographic variables on reproductive output can be assessed with minimal confounding ecological variation compared to studies of wild populations by comparing groups of different size and composition that live in the same ecological habitat. Finally, there is significant variation in the age of high-ranking males on the island, which may be facilitated by food provisioning that may provide opportunities for older males to maintain high-ranking positions more frequently than they would be able to under more ecologically stressful conditions. This pattern may be further facilitated by the succession rank system in rhesus macaques, in which males typically achieve high dominance status based on their tenure in a group^27^ (but see^28^) rather than by challenges to other males^29^.

Importantly, dominance hierarchies are not always stable and variation in group dynamics could also impact male reproductive success. Periods of social instability in nonhuman primate groups can have behavioral and physiological consequences for males in a dominance hierarchy^30–34^. These consequences could impact a male’s ability to successfully reproduce. Social instability has been associated with energetic stress in male primates (rhesus macaques^33^, baboons^30,32^), which could lower a male’s ability to compete for mates or produce viable offspring. Additionally, males may have to focus more time on aggressive interactions with other males instead of mating effort. For example, we have previously documented variation in this population in the use of behavioral displays—males in stable groups display in mating contexts more often than males in groups with unstable hierarchies and males in groups with unstable hierarchies instead display in aggressive or dominance interactions with other males^34^.

Here, we examine variation in the reproductive success of high-ranking male rhesus macaques on Cayo Santiago and the potential factors influencing this variation to determine if reproductive senescence is present in this species. We compare the number of offspring sired by high-ranking males in 2013 in relation to their age, rank, and the stability of their social group, as well as their mating effort. We also investigated how their reproductive output for the study year compared to their lifetime reproductive success prior to that year. Although we have data on the total number of surviving offspring males have sired over their lifetime, we do not have any behavioral data (including mating behaviors and assessments of dominance rank) outside of the study period. Therefore, we present detailed analyses of variation in male reproduction for the 2013 study period and compare this to what is known from the genetic database for the study subjects. Overall, we expected top ranking males in stable social groups (where their dominance rank did not change during the study) to produce more offspring than high-ranking males in groups with unstable hierarchies and that there would be a positive correlation between mating effort (days spent consorting, number of mounts, and number of ejaculations observed) and the number of offspring sired in 2013. We considered deviations from these patterns for older males as evidence of reproductive senescence. Specifically, we predicted that if reproductive senescence impacts male reproductive success in rhesus macaques, then (1) older high-ranking males of stable social groups would have lower reproductive output than younger high-ranking males in stable social groups, (2) older high-ranking males would have fewer offspring in recent years than over their lifetime, and (3) the mating behaviors of older males would not be correlated with the number of offspring produced in that year. Our results provide novel evidence of reproductive senescence in high-ranking male nonhuman primates.

## Methods

### Site and subjects

The island of Cayo Santiago in Puerto Rico is home to a colony of free-ranging rhesus macaques that was first established in 1938 with approximately 400 wild-caught animals from India^35^. At the time of this study, the population had grown to over 1,200 individuals, which had naturally divided themselves into 9 social groups. The monkeys are provisioned with water and commercially available monkey chow. Records of all births and deaths have been maintained since 1956 and all individuals on the island currently have been genotyped to determine both maternal and paternal lineage^36^.

As previously described^34^, the subjects of this research were 21 adult males that were the highest ranking individuals in the 9 social groups, as determined at the beginning of the study by outcome of fights, access to food, displacement events, fear grins, and avoidance behaviors^37^. Dominance rank was subsequently reassessed throughout the study through ad libitum observations, and five of the nine groups were considered unstable because of changes in the dominance ranks of the top-ranking males in these groups. Two of these unstable groups were small groups that had formed within the year prior to this study, stayed on the periphery of the larger group from which they had split-off, and were not considered independent from the larger group during parts of the study period. For this reason, only three males were studied from these two groups combined. These males were the highest ranking within their small groups and were therefore classified as top-ranking (because of their high rank within the newly formed groups), but none of the individuals were identified as alpha because they were submissive to the higher-ranking individuals in the larger group to which they were peripheral. The other three groups with unstable hierarchies had major changes in the rank of the highest ranking males throughout the study, resulting in rank changes for the alpha, beta (second highest-ranking males in a group), and gamma (third highest-ranking male in a social group) males in those groups. The other four groups had stable dominance hierarchies throughout the behavioural study period of 2013. The rank of an individual within a stable group or membership in an unstable group is being referred to as “rank stability” within this paper (see Supplementary Table 1 for further information about the study subjects). Due to the comparative nature of this study, including the need to gather detailed behavioral and reproductive data on each subject with subjects ranging across every group on the island, it was not possible to include more subordinate males. Given that high-ranking male primates generally have access to mating partners, prioritizing data collection from these individuals provided the best opportunity to measure variation in reproductive success for males who were actively engaging in mating behaviors. The investigation was approved by the IACUC of the University of Puerto Rico, Medical Sciences Campus (protocol No. A0100108). All methods were performed following the relevant regulations and guidelines.

### Behavioural observations

Behavioural data were collected 5 days a week from approximately 0700 to 1430 during the 2013 mating season from mid-February to mid-July. The rhesus macaques on Cayo Santiago have distinct breeding and birth seasons, so behavioral observations were collected during the breeding season and data about the offspring produced during the birth season were gathered from the database. Each male was observed for a 10-min focal follow on a rotating basis at least every other day during the week. These follows were extended to 60 min if the male was engaged in a sexual consortship with a female. Consortships are prolonged associations between the male and female where the pair travels together, has high rates of affiliative behaviors, and mates during one or multiple mounting series. Scans were taken at the start of the focal and every 2 min after until the 10 min were complete or until the 60 min were complete. During consortships, all mating behaviors were recorded, including mounting and ejaculation.

### Paternity data

Genetic paternity data were gathered from the long-term database managed by the Caribbean Primate Research Center. Through this database, we were able to determine the number of offspring each focal male sired in 2013, as well as how many offspring he sired prior to 2013. We used both the total counts of offspring up to 2013, as well as the average number of offspring per year during a male’s “adult” life by averaging the total number of offspring over the number of years older than four the male was. This method was to account for different ages, and used five as the earliest possible age of reproduction for males on Cayo Santiago, based on the earliest age of first reproduction for males in this population. Offspring paternity was determined when they were yearlings, meaning that infants who died prior to this sampling period would not have been included in the calculations. In 2013, there were 33 infant deaths and seven stillbirths. Infant mortality was 13% of all infants born that year. We cannot account for who sired these offspring.

### Statistical analyses

Generalized linear mixed models fitted with a Poisson error structure were used to examine how male age, group stability, and the interaction of these two factors impacted the number of offspring produced, as well as the interaction between a male’s age and the number of days he spent consorting on the number of offspring he sired that year. We treated group ID as a categorical random effect in the model. To compare the number of offspring a male had sired prior to 2013 and in 2013 to their rank stability, we used Kruskal–Wallis tests. In these analyses, there were four categories of males: (1) alpha males in stable groups, (2) beta males in stable groups, (3) gamma males in stable groups, and (4) high-ranking males in groups with unstable hierarchies. The dataset included three males that were seven or eight years old, lived in groups with unstable hierarchies, and sired no offspring. This age category is the average age for first reproduction in male rhesus macaques, but many males do not produce their first offspring until after this age^38^. Given this pattern for age at first reproduction and that there were no males from the stable groups that were this young, we removed these three males from additional analyses on reproductive success. A Spearman correlation coefficient was calculated for male age (males > 8 years old) and number of offspring sired in 2013, as well as different indicators of male mating effort (days spent consorting, number of mounts, and number of ejaculations) and the number of offspring spired in 2013. All analyses were conducted in SAS version 9.4 (SAS Institute Inc., Cary, NC, USA). A *p* value < 0.05 was considered significant.

## Results

### Age and male reproduction in the study period

**Figure 1:**
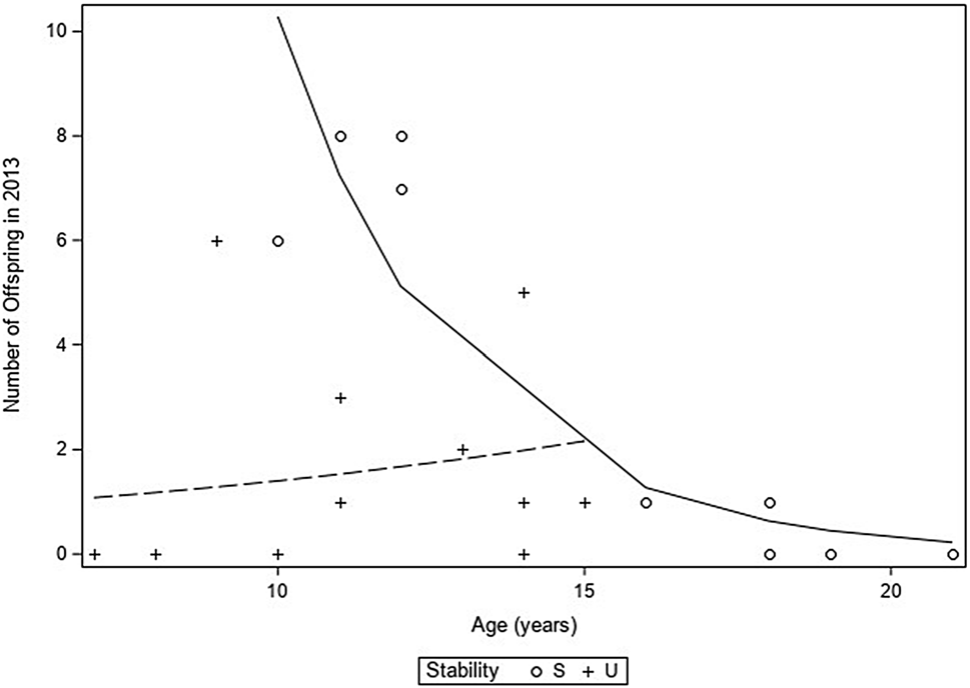
The age of high-ranking males living in stable groups (S) is negatively correlated with the number of offspring they sired during the 2013 breeding season, particularly after the age of 15, but this pattern does not apply to males in unstable hierarchies (U) between the ages of 9 and 15.

Using a generalized linear mixed model, we found that during the 2013 breeding season, group stability (*p* = 0.0101) and the interaction of group stability and male age (*p* = 0.0214) significantly accounted for the number of offspring produced that year (Table 1). In stable groups, older males had fewer offspring, but in unstable groups, the number of offspring did not decrease with age. Importantly, the oldest males in our study were all in the groups with stable dominance hierarchies. In our dataset, there were three males younger than age nine. These three males (ages seven and eight) were part of unstable groups and sired no offspring. Given the young age of these males and that there were no males from the stable groups that were this young, we removed these three males from additional analyses. Using the 18 remaining subjects that were nine years old and older, we found a significant negative correlation between age and the number of offspring sired in 2013 (*ρ* = -0.58, *p* = 0.0117). The alpha, beta, and gamma males in stable groups were driving this relationship while males in groups with unstable hierarchies had highly variable reproductive rates (Fig. 1). Importantly, all of the males in the groups with unstable hierarchies were age 15 and younger whereas high-ranking males in stable groups ranged in age from 10 to 21 years. Older males in stable groups had fewer infants, if any, in 2013 than the younger males in those groups (Fig. 1).

**Table 1:** The effect of age and stability on the number of offspring produced by each male (n = 21) in 2013.

Covariates	Estimate	SE	DF	Chi-square	*P* value
Age	0.0869	0.1006	1	1.25	0.2632
Stability (S)	6.3470	1.8096	1	6.61	0.0101
Age*stability (S)	− 0.4353	0.1169	1	5.30	0.0214

**Figure 2:**
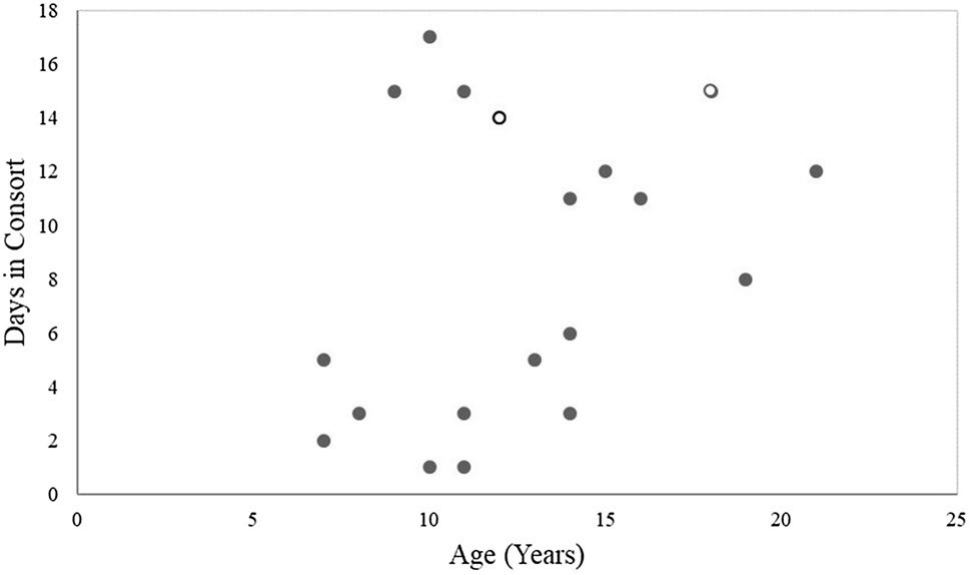
Older males did not spend less time in consortships with females than other males. This graph shows the number of observation days a male was observed consorting with a female for each subject (N = 21). The black dots each represent one male, and the white dots each represent two males (i.e. there were two 11-year-olds that each were observed consorting on 14 days of the study period and two 18-year-olds who were each observed consorting for 15 days each). The oldest males in the study consorted with females on many of the days they were observed, as shown in this figure.

**Figure 3:**
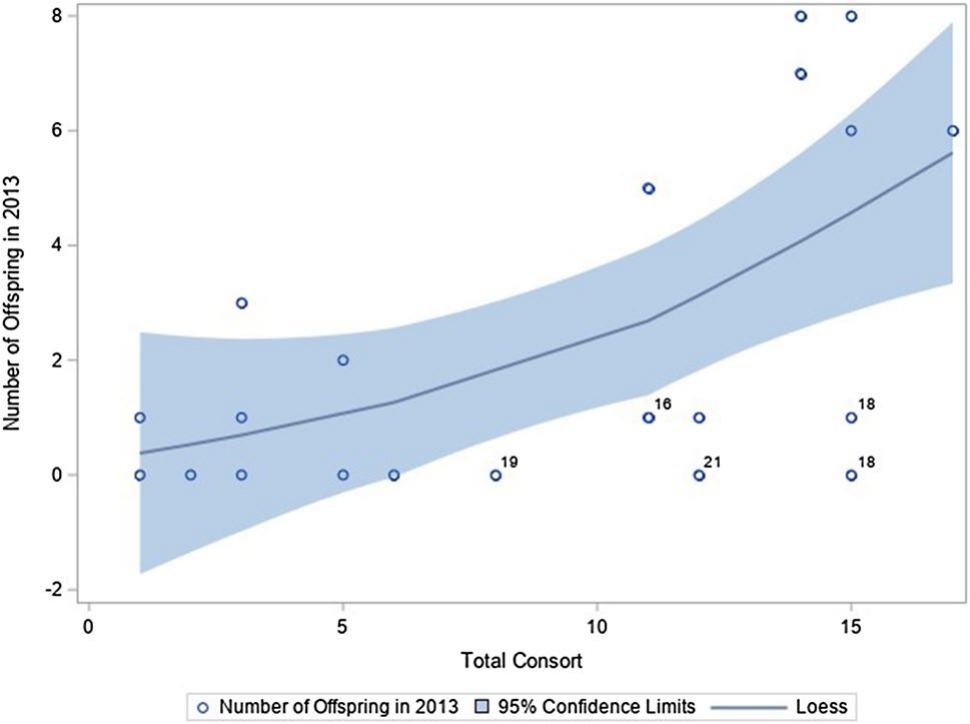
There was a positive association between the number of days males spent in consortships and the number of offspring they sired that year, but outliers included the older males from stable groups (the ages of these males over 15 years old are indicated). The LOESS (locally weighted smoothing) line created from the regression analysis uses a smoothing value of 1 and the 95% confidence limits are shown with the shading.

Older males (males > 15 years old) consorted with females on many of the days they were observed (Fig. 2). Across all males, there was a significant positive relationship between the number of days a male was observed consorting (*ρ* = 0.49, *p* = 0.0242; Fig. 3), the number of times they were observed mounting a female (*ρ* = 0.57, *p* = 0.0072), and the number of times they were seen ejaculating (*ρ* = 0.52, *p* = 0.0159) with the number of offspring sired in 2013, but deviations from this pattern were primarily the males over 15 years old from stable groups. Using a generalized linear mixed model, we found that both the number of days consorting (*p* = 0.0008) and the interaction between the number of days consorting and the male’s age (*p* = 0.0387) were significantly associated with variation in the number of offspring sired in 2013 (Table 2). This interaction is such that the amount of time a male spends consorting with females impacts the number of offspring they produce until a certain age when the likelihood of producing an offspring becomes very low regardless of mating effort.

**Table 2:** The relationship of male (n = 21) age and number of days consorting to the number of offspring produced in 2013.

Covariates	Estimate	SE	DF	Chi-square	*P* value
Age	0.1484	0.1502	1	0.99	0.3198
Consort	0.4310	0.1397	1	11.32	0.0008
Age*consort	− 0.0254	0.0126	1	4.28	0.0387

Two of the oldest males in this study (18 years and 21 years) were both alpha males of stable groups. They had produced the most offspring prior to 2013 out of all the focal males (52 and 47 offspring, respectively). They both were observed in consortships frequently with females on days they were observed (63% and 41% of observation days, respectively, compared to, for example, 60% and 48% for the two males who produced the most offspring in 2013 out of the study subjects). The 18-year-old had the highest number of observed ejaculations in the study. Yet, these males produced the fewest offspring (1 and 0, respectively) out of all the alpha males in the study. The other two alpha males of stable groups were 10 and 11 years old and produced 6 and 8 offspring, respectively, in 2013.

In groups with old alpha males, the ability to sire a high number of offspring in 2013 moved down the dominance hierarchy to other high-ranking males (Supplementary Table 1). The beta male in the group with the 18-year-old alpha male had 7 offspring. The gamma male in that group was 19 years old and did not produce any offspring. The beta male in the group with the 21-year-old alpha was also old (18 years) and also did not produce any offspring. However, the gamma male in that group was 12-years-old and had 8 offspring (tied with one of the alpha males for the most offspring produced in the study).

### Male reproductive success prior to the study period

**Figure 4:**
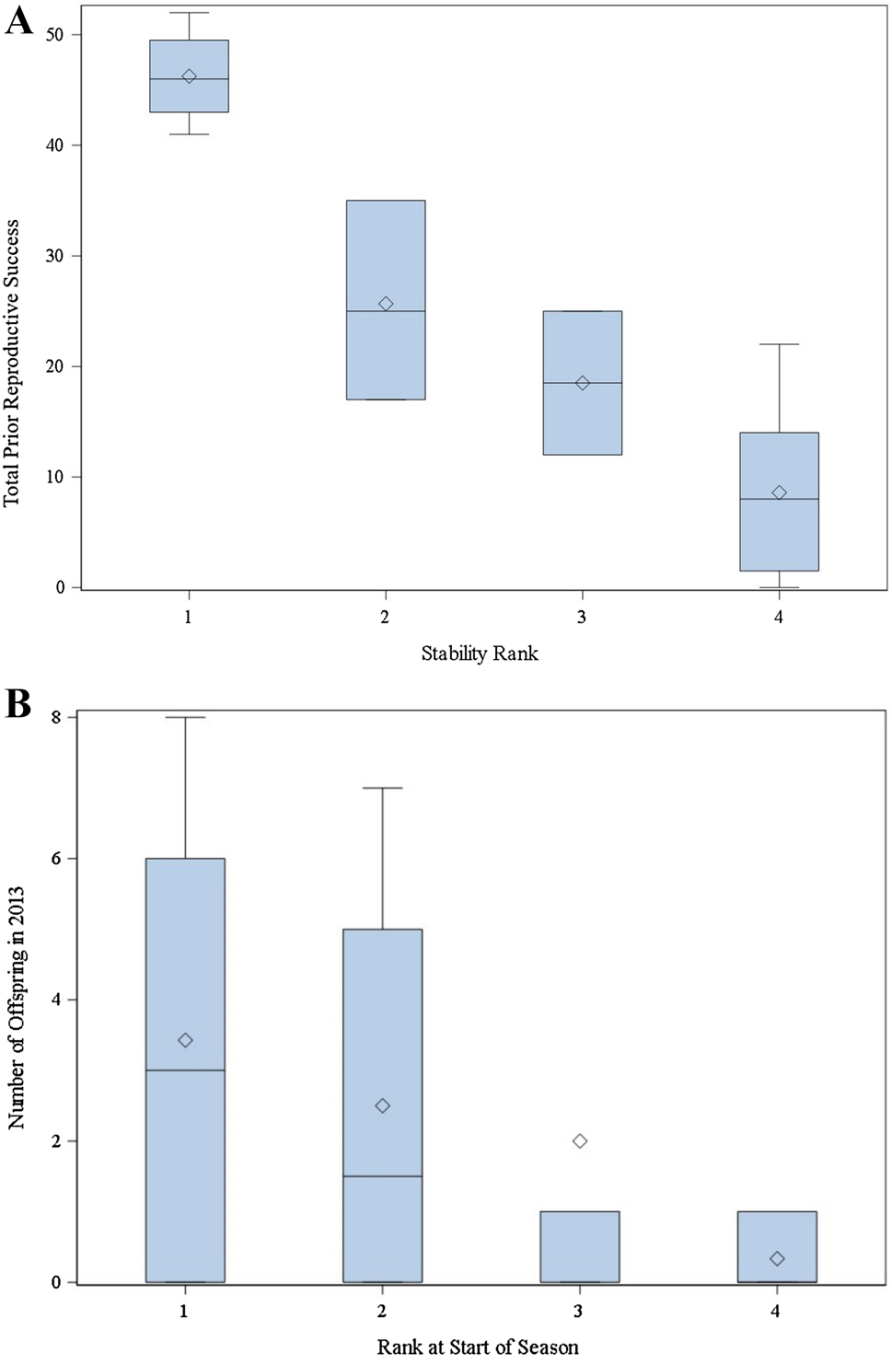
Alpha (1), beta (2), and gamma (3) males of stable groups and males in unstable hierarchies (4) differ in the total number of offspring they sired up to the 2013 mating season (**A**), but these rank categories did not predict reproductive success in the 2013 mating season (**B**). These boxplots show the variation in the mean (diamond) and median (line).

The total number of offspring a male sired leading up to the 2013 breeding season differed significantly based on the males’ rank and membership in a stable or unstable group (χ^2^ = 13.61; *p* = 0.0035) with alpha males of stable groups, in particular, having more offspring than males in groups with unstable hierarchies (Z = 2.91; *p* = 0.0188; Fig. 4A). To account for variation in age (and thus the number of years a male had to reproduce), we also compared the average number of offspring a male produced per year since the age of 5 and found the same pattern (χ^2^ = 10.03; *p* = 0.0183) with alpha males of stable groups, in particular, having more offspring than males in groups with unstable hierarchies (Z = 2.79; *p* = 0.0270). However, there was no difference in the number of offspring males of different rank categories sired during the 2013 breeding season (χ^2^ = 2.41; *p* = 0.4922; Fig. 4B). While we cannot account for variation in rank and mating effort in other years, we did also find that these older males with high lifetime reproductive success had declining reproductive output in the years immediately prior to this study (as they aged further away from 15). The 21-year-old alpha male had no offspring in both years prior to the study period—at ages 19 and 20. The 18-year-old alpha male that had one offspring during the study period also had one offspring per year at ages 16 and 17. The 18-year-old beta male had 2 offspring at age 16 and 1 offspring at age 17.

## Discussion

Through this study of the reproductive output of high-ranking male rhesus macaques across all the groups on Cayo Santiago in 2013, we provide evidence of variation in male reproductive success across males of different rank stabilities and males of different ages. High-ranking male rhesus macaques in stable groups had high lifetime reproductive success prior to 2013. Given that male dominance ranks are not regularly assessed in every group on Cayo Santiago, we cannot comment on the directionality of that relationship or changes in rank status before or after the study period. High-ranking males in stable groups may be able to secure long tenures at top ranked position and therefore sire many offspring over the years or males that sire many offspring may be able to rise to high positions within the dominance hierarchy. Although we cannot assess the directionality of that relationship, it is clear that a male’s rank stability during the 2013 mating season was significantly associated with variation in the number of offspring that male had prior to 2013. For males under 16 years old, this pattern was maintained in 2013, with those high-ranking males in stable groups having high reproductive output; however, older males had significantly lower reproductive output, particularly those males aged 18 to 21 years. Males of this age make up a small percentage of the population on Cayo Santiago, with the life expectancy at birth of males being 14.4 years and the maximum lifespan of males being 29-years-old^39^. Hoffman et al.^17^ found a similar result for female rhesus macaques on Cayo Santiago with poor infant survival for older females, especially those who were 18 years and older.

Patterns of male rank and lifetime reproductive success in the high-ranking males on Cayo Santiago are consistent with findings from other species of primates (*Papio cynocephalus*^21^, *Pan troglodytes verus*^40^, *Pan troglodytes schweinfurthii*^41^) and in previous studies of this population^42,43^. The findings are also consistent with previous work showing a positive association between dominance rank and reproductive interactions with females around ovulatory periods in this population^44^. The patterns also fit with the predictions of the priority of access model, which posits that the number of offspring sired by a male will be influenced by the male’s rank, his access to females, and the number of competitors present^2^. The deviation from these patterns for the older high-ranking males in the group are important.

In other species, declines in reproductive output for older males have been associated with a loss of dominance status^5,45^, loss in attractiveness^46^, and/or loss in body condition^5^ leading to a decline in mating activity for older males^5,45–47^. But the older males in this study maintained high rates of mating behaviors while still experiencing the same decline in reproductive output. Additionally, previous studies showed that male body condition remained stable in adult male rhesus macaques (including those over the age of 15) even as reproductive output declined, with males between 17 and 24 years old producing no more than one infant per year^38^, and older males had high measures of immune function and low oxidative stress^48^. Furthermore, aging rhesus macaque males do not have erectile issues^16^.

Previous studies of reproductive success in older male macaques have had variable results. In Japanese macaques (*Macaca fuscata*), old males that were central in the study group did not sire any offspring^49^. Yet in Barbary macaques (*Macaca sylvanus*), there was no decline in reproduction for older males, with males aged 18 and older siring offspring^50^. In our study, we found that males aged 9 to 14 produced the most infants during the study period. Previous studies of the Cayo Santiago rhesus macaques found similar results with the highest number of offspring being sired by males that were 9 to 11 years old^24^ or 7 to 12 years old^51^ and very few offspring being produced by the oldest males in the study (18 and 19 years old^51^), but these studies did not show that age-related differences existed despite older males continuing to be high-ranking and having high rates of mating behaviors.

In previous studies of changes in rhesus macaque reproductive function over age, older males were found to exhibit fewer sexual behaviors, including mounting and ejaculation^22^; however, we found that in this study, older high-ranking males maintained a high rate of sexual activity. Older males that produced no surviving offspring or only one surviving offspring had comparable rates of consorting with females, mounting females, and ejaculating during mounts to their younger counterparts. Given the patterns of mating effort by these males and the evidence of previous fertility and reproductive success for these individuals, our results suggest that the low reproductive output of older high-ranking males is a result of post-copulatory reproductive senescence. These results add to a sparse literature on male post-copulatory reproductive senescence in free-ranging animals. Lemaître and Gaillard^25^ argue that this lack of age-specific data on reproductive success prevents research on the factors impacting reproductive senescence. Reed et al.^52^ found reduced reproductive output for male seabirds, common guillemots (*Uria aalge*), in the final year of life. Similar to our findings in this study, Wroblewski et al.^41^ found that older male (> 19 years old) chimpanzees were less successful at producing offspring than younger males (15 to 19 years old), even as the rank of those older males continued to rise. Our results provide some of the only evidence of lower reproductive output for older males despite maintaining high rates of mating behaviors. Additionally, our results indicate that behavioral observations of mating behaviors are not reliable indicators of male reproductive output and should not be used as such.

The relatively large testes of male rhesus macaques compared to their body size suggest that sperm competition^53^ is likely an important aspect of reproductive success in this species^54,55^. Female rhesus macaques consort with multiple males during an estrous cycle^56,57^. Additionally, sneaky copulations are a successful mating strategy in macaques (rhesus macaques^58^, Japanese macaques^59^). Although this mating strategy is not as successful as consorting for siring offspring in male rhesus macaques^58^, it does introduce additional opportunities for sperm competition. Alpha male rhesus macaques are unable to fully monopolize females because of the high degree of estrous synchrony^43,60^, and they may benefit from being tolerant of other males in their group mating because it may lead to longer tenure length for those alpha males. For example, dominant male geladas (*Theropithecus gelada*) that were tolerant of other males in their groups had larger groups of females and longer tenure lengths compared to single-male units; thus, they had reproductive benefits even though they were sharing reproductive opportunities with other males each year^61^. Given the patterns of multiple matings by females, it is likely that sperm competition plays an important role in paternity in rhesus macaques. Furthermore, these patterns highlight the potential importance of female mate choice in rhesus macaques. Future research should investigate the role of females in biasing paternity against males over 15 years of age.

It is possible that the older males in our study may have had reduced sperm quantity or quality. Sperm depletion may be more noticeable in highly-ranked males who have more mating opportunities, and older males may no longer be physiologically capable of producing as much sperm as necessary for these high rates of mating behaviors^41^. For example, in a study of domestic fowl (*Gallus gallus domesticus*), older male ejaculate had fewer sperm than young males and those sperm had lower swimming velocity, which meant that young male ejaculate had a strong fertilizing advantage over older male ejaculate^62^. Our previous work suggests that high-ranking male rhesus macaques, especially alpha males, experience significant energetic stress during the breeding season, particularly at the beginning of the season when the maximum number of fertile females are available^63^. The toll of this stress and of investing effort into mate guarding and behavioral displays during mate guarding^34^ may have reproductive costs for older males. In men, age-related declines in sperm production and quality have been reported^8,9,11–13^, but there are contradictory reports (see^10,12^) and variation cross-culturally^9^. Future studies should explore variation in sperm quantity and quality over the lifetime. Given the difficulty of measuring ejaculate quality directly, examining changes in testes size may provide a proxy for understanding reproductive senescence in older male rhesus macaques, especially given that Bercovitch^64^ found that high-ranking males had the largest testes during the mating season.

In addition to decreased sperm quality or quantity, low reproductive output for older males may be the results of low infant survival (similar to the pattern shown in females^17^). Given that we could not account for the paternity of the 33 infants that died and the 7 stillbirths from the study year, we cannot determine how many of those infants were fathered by our study subjects. It is possible that older males have low infant survival or low sperm quality or both. Ultimately, these issues still result in reduced reproductive success for older males, indicating post-copulatory reproductive senescence.

Evidence of mechanisms of age-specific rates of reproduction are important for understanding life-history evolution, yet few studies exist on this topic. Most studies of reproductive skew focus on a male’s ability to monopolize females and female mate choice, but do not examine differences in male reproductive physiology over age in relation to skew. Future studies should incorporate measures of reproductive senescence in understanding variation in male reproductive success over their lifetime, including examining long-term cost of early reproductive effort (see^65^). The timing of reproduction in male rhesus macaques is influenced by variation in the serotonin transporter gene^51^, and future studies should investigate differences in reproductive senescence in relation to genotype. Importantly, we were not able to compare males of all ranks within this study. Future studies should also compare variation in reproductive success for males in other rank categories.

Our results provide important considerations for understanding male reproductive senescence across primates and potential evolutionary underpinnings of reproductive senescence in humans. Infertility is a worldwide clinical problem for human health and affects 8–12% of couples^9^. To add to this problem, there has been a significant increase in parental age in recent years^12^. As with studies of nonhuman animals, most studies of reproductive senescence in humans have focused on women. Studies of age-related changes to men’s sperm have provided contradictory evidence^10,12^. And cross-cultural variation in these changes make the issue difficult to understand through human studies^9^. Furthermore, most studies in reproductive senescence use a cross-sectional approach to examine variation in reproductive functioning. Here, we provide evidence of decline in reproductive activity within individuals. Future studies should continue to gather these long-term data on variation in male reproductive health and how social and physiological factors can impact a male’s ability to sire offspring.

## Supplementary information

Supplementary Information.
