# Supplementary material for: Age negatively impacts reproduction in high-ranking male rhesus macaques on Cayo Santiago, Puerto Rico
Source: Sci Rep. 2020 Aug 3;10:13044. doi: 10.1038/s41598-020-69922-y (PMC7398901; doi:10.1038/s41598-020-69922-y)
Supplement: Supplementary file 1 — Supplementary Information. [file 41598_2020_69922_MOESM1_ESM.docx]

**Supplemental Table 1: Focal Subject Data**

| **Male** | **Group** | **Stability** | **Age** | **Stability Rank** | **2013 Offspring** | **Total Offspring** | **Offspring Per Year *** | **Consort Days** | **Mounting Days** | **Observed Ejaculation** |
| --- | --- | --- | --- | --- | --- | --- | --- | --- | --- | --- |
| Male 1 | Group 1 | S | 18 | 1 | 1 | 52 | 3.71 | 15 | 4 | 3 |
| Male 2 | Group 1 | S | 12 | 2 | 7 | 35 | 4.38 | 14 | 3 | 1 |
| Male 3 | Group 1 | S | 19 | 3 | 0 | 12 | 0.80 | 8 | 1 | 1 |
| Male 4 | Group 2 | U | 9 | 4 | 6 | 12 | 2.40 | 15 | 2 | 2 |
| Male 5 | Group 2 | U | 14 | 4 | 5 | 15 | 1.50 | 11 | 3 | 2 |
| Male 6 | Group 2 | U | 15 | 4 | 1 | 19 | 1.73 | 12 | 3 | 1 |
| Male 7 | Group 3 | U | 11 | 4 | 3 | 22 | 3.14 | 3 | 1 | 1 |
| Male 8 | Group 3 | U | 13 | 4 | 2 | 10 | 1.11 | 5 | 1 | 1 |
| Male 9 | Group 3 | U | 14 | 4 | 1 | 13 | 1.30 | 3 | 2 | 0 |
| Male 10 | Group 4 | U | 14 | 4 | 0 | 6 | 0.60 | 6 | 2 | 1 |
| Male 11 | Group 4 | U | 7 | 4 | 0 | 0 | 0.00 | 5 | 1 | 0 |
| Male 12 | Group 4 | U | 8 | 4 | 0 | 0 | 0.00 | 3 | 4 | 2 |
| Male 13 | Group 5 | U | 11 | 4 | 1 | 2 | 0.29 | 1 | 0 | 0 |
| Male 14 | Group 6 | U | 10 | 4 | 0 | 3 | 0.50 | 1 | 1 | 1 |
| Male 15 | Group 6 | U | 7 | 4 | 0 | 1 | 0.33 | 2 | 1 | 0 |
| Male 16 | Group 7 | S | 11 | 1 | 8 | 41 | 5.86 | 15 | 9 | 2 |
| Male 17 | Group 7 | S | 16 | 2 | 1 | 17 | 1.42 | 11 | 4 | 1 |
| Male 18 | Group 8 | S | 10 | 1 | 6 | 45 | 7.50 | 17 | 8 | 1 |
| Male 19 | Group 9 | S | 21 | 1 | 0 | 47 | 2.76 | 12 | 0 | 0 |
| Male 20 | Group 9 | S | 18 | 2 | 0 | 25 | 1.79 | 15 | 0 | 0 |
| Male 21 | Group 9 | S | 12 | 3 | 8 | 25 | 3.13 | 14 | 4 | 2 |

*Average based on number of total offspring produced prior to the study period divided by male’s age minus 4 (e.g. the average offspring produced each year from age 5 to the time of the study).
